# Is Science for Us? Black Students’ and Parents’ Views of Science and Science Careers

**DOI:** 10.1002/sce.21146

**Published:** 2015-02-18

**Authors:** LOUISE ARCHER, JENNIFER DEWITT, JONATHAN OSBORNE

**Affiliations:** ^1^ DEPS, King's CollegeLondonUK; ^2^ Graduate School of EducationStanford UniversityStanfordCA94305USA

## Abstract

There are widespread policy concerns to improve (widen and increase) science, technology, engineering, and mathematics participation, which remains stratified by ethnicity, gender, and social class. Despite being interested in and highly valuing science, Black students tend to express limited aspirations to careers in science and remain underrepresented in post‐16 science courses and careers, a pattern which is not solely explained by attainment. This paper draws on survey data from nationally representative student cohorts and longitudinal interview data collected over 4 years from 10 Black African/Caribbean students and their parents, who were tracked from age 10–14 (Y6–Y9), as part of a larger study on children's science and career aspirations. The paper uses an intersectional analysis of the qualitative data to examine why science careers are less “thinkable” for Black students. A case study is also presented of two young Black women who “bucked the trend” and aspired to science careers. The paper concludes with implications for science education policy and practice.

## BACKGROUND

There is a widespread concern among governments and policy makers in Western developed nations (e.g., ACOLA, 2013; US President's Council of Advisors on Science and Technology, 2010; Danish EU presidency, 2012) that more needs to be done to improve—to increase and widen—participation in postcompulsory science, technology, engineering, and mathematics (STEM). This is particularly the case for the physical sciences, where the “typical” graduate remains White, male, and middle class (e.g., AAUW, 2010; Smith, 2010a, 2010b, 2011), despite decades of interventions aimed at broadening the profile of scientists in university and beyond. The rationale for increasing participation in STEM has predominantly been framed in terms of the importance of ensuring an appropriately qualified STEM workforce to fulfill the demands of national economies. Yet STEM participation is also important for the “good society” (e.g., Millar & Osborne, 1998), and there is a social justice case to be made for the value and need for all citizens to be to able to participate in STEM, to be literate, and shape STEM developments in society.

Figures from the United Kingdom and United States indicate that, in general, those from minority ethnic backgrounds are underrepresented in physical science degrees and are less likely to work in science, engineering, and technology professions (Adamuti‐Trache & Andres, 2008; Ceci, Wiliams, & Barnett, 2009; Hills et al., 2010; Smith, 2011). But participation rates also vary between different ethnic groups. Some groups, notably those of White, Indian, and Chinese heritage, are more likely to participate in postcompulsory science. While those from Black,^1^ Pakistani, and Bangladeshi backgrounds tend to be underrepresented (Elias & Jones, 2006; Malcom, Van Horne, Gaddy, & George, 1998; Pearson & Bechtel, 1989).

Although the underrepresentation of minority ethnic science students in the physical sciences at university level has previously been attributed in part to low attainment (Royal Society, 2008), this does not fully explain differential patterns of participation (Strand, 2007, 2009). Even when appropriately qualified, minority ethnic students are more likely to choose *not* to study the physical sciences at university than appropriately qualified White students. For instance, in the United Kingdom, British Indian students are more likely than other groups to have the necessary qualifications to enable them to study for a physics degree at university, but they are proportionally less likely to enter such courses (Jones & Elias, 2005). As Jones and Elias (2005) further point out, this pattern continues into the workplace, where men and those from White backgrounds are proportionally overrepresented among STEM professionals, compared to national population percentages.

To date, considerable research attention, resources, and interventions have focused on gender, attempting to improve the representation and participation of women and girls in science. Comparatively, less attention has been given to improving participation among underrepresented ethnic minority groups and working‐class students. This paper addresses issues of “race”/ethnicity, with a particular focus on the identities and aspirations of Black students, although we also attempt to bring an intersectional lens to bear on the data to uncover ways in which ethnicity interacts with gender and social class to make science aspirations less “thinkable” (conceivable and achievable) for Black students.

### Black Students' Participation in Science

Wider research on the reasons for Black students' more general underattainment in education has pointed to a complex mixture of factors, underpinned by pervasive and institutionalized racism within society. There is a substantive body of research evidence pointing to the pervasive and pernicious effects of the lower expectations that teachers have been found to express for minority ethnic students (e.g., Ball, 2003; Crozier, 2009; Crozier & Davies, 2008; Gillborn & Mirza, 2000; Gillborn, 2001; Wright, Standen, & Patel, 2010). Teachers’ (often unconscious) stereotyping and lower expectations have been found to be differentiated, with differing expectations stereotypically expressed for different ethnic groups—although these have also been found to be consistently lower for Black students than for those from Indian and Chinese backgrounds (Archer, 2008; Archer & Francis, 2007).

In the case of Black students’ engagement with and participation in science, research suggests that institutional racisms are also underpinned by the sociohistoric exclusion of minority ethnic groups from scientific knowledge in Western societies (Baker, 1998). The challenges experienced by minority ethnic students within the science classroom have been powerfully documented within schools (e.g., Atwater, 2000; Brickhouse & Potter, 2001; Carlone & Johnson, 2007; Carlone et al., 2011; Rascoe & Atwater, 2005) and higher education (e.g., Atwater & Simpson, 1984; Malone & Barabino, 2009; Ong, 2005; Russell & Atwater, 2005; Seymour & Hewitt, 1997; Vining‐Brown, 1994).

For instance, Atwater (2000) points to the symbolic violence experienced by Black girls within science classrooms in which “White is the norm,” and Malone and Barabino (2009) discuss how minority ethnic students report feeling isolated and marginalized within science classrooms. Black students have also been found to be among those most likely to report finding it hard to position themselves, and to be seen by others, as “properly” or authentically scientific (Carlone & Johnson, 2007).^2^ In the United States, researchers have been developing understandings of how “race,” class, and gender intersect to shape urban working class girls’ engagement with science (e.g., Brickhouse et al., 2000). Brickhouse and Potter (2001) show how, for young women of color, “the experience of marginalization can make membership in a school science community impossible or undesirable” (p. 965). Science teachers have been shown to play a key role in constraining or facilitating Black students’ ability to perform authentic scientific identities (Calabrese Barton, Tan, & Rivet, 2008; Calabrese Barton & Tan, 2009). Research has also, importantly, pointed to the ways in which Black students’ engagement with science can be substantively improved through the adoption of culturally respectful and “caring” approaches (e.g., Parsons, 2008) and/or approaches which validate and utilize urban youths’ own cultural resources (e.g., Elmesky, 2001, see also Basu, Calabrese Barton, & Tan, 2011; Tan & Calabrese Barton, 2012).

It has been argued that interactions and subject positions within the science classroom are underwritten by wider social relations, in which science is dominantly constructed as White, male, and middle class (Harding, 1998; Losh, 2010). Hanson (2007, 2009) argues that the culture of science is particularly hostile to Black students, creating a “chilly climate” for Black girls and women, who can experience both racism and sexism within science environments (Malcom et al., 1998).

As a result, and as Carlone and Johnson (2007 p. 1207) put it, “the institutional and historical meanings of being a scientist’ actually means ‘being a white male,’” a message which is reinforced within many science classrooms (Atwater, 2000). Carlone and Johnson's longitudinal study of women scientists of color highlights how minority ethnic science students and professionals can feel alienated by the culture of science in higher education (see also Atwater & Simpson, 1984), requiring them to work twice as hard, to not only achieve and succeed, but also to “fit in” and attain a sense of authenticity and belonging (Carlone & Johnson, 2007). This identity work can be extensive and profound, requiring, for example, minority ethnic women scientists to carefully negotiate ways of dressing, speaking, and behaving (Ong, 2005). Carlone and Johnson's (2007) research also indicates that Black women are disproportionately likely to report experiences of alienation and invisibility and to feel that their science career trajectories had been disrupted. The women in their study who reported such experiences

Felt overlooked, neglected, or discriminated against by meaningful others within science …they felt that established members of their science departments recognized them not as science people but, instead, as representatives of stigmatized groups. They perceived that their behaviors, or even just their appearance, triggered racial, ethnic or gender recognitions that overwhelmed their chances of being recognized as good science students. (Carlone & Johnson, 2007, p. 1202)

As Scantlebury and Baker (2007) point out, science education research focusing on both race and gender, as a combined unit of analysis, is a relatively recent phenomenon. In their recent literature review, Pinder and Blackwell (2013) argue that there has been woefully little research on Black girls and science education, and call for more sustained work to address simultaneous interactions of race and gender. Buck and Quigley (2013) also call for science education research to explore even more complex intersections.

This paper attempts to contribute to the emerging and growing critical scholarship on intersections of “race,” gender, and social class in relation to diverse students’ engagement with science. In particular, we ask why are Black students less likely to express aspirations for careers in science? What makes science careers less “thinkable” (conceivable and achievable)? And what enables some students to buck the trend and “aspire otherwise”?

## THEORETICAL FRAMEWORK

Our theoretical approach draws on (i) feminist, postcolonial, and “intersectional” poststructuralist theorizations of identity as a tool for understanding students’ identifications with science and (ii) a Bourdieusian‐inspired conceptualization of “science capital.” Our approach understands identity as nonessentialized, fluid, contested, and produced through discourse (Anthias, 2001; Burman & Parker, 1993; Gee, 1996). As Hall (1990, p. 222) writes, identities are “always in process,” being constituted within and through discourse and relations of power (Foucault, 1978). Following the feminist poststructuralist work of Butler, we see identities as discursive and embodied “performances” (Butler, 1990, 1993), which are never achieved but must be continually done and redone. In other words, gender is a socially constructed “act” that is produced through discursive and bodily practices—it is not simply the product of a person's sex and, in this sense, is a constructed (not “natural”) product of particular (sexed, racialized, classed) bodies. In short, gender is not something you “are” or “have” but rather is something that you continually “do” (perform) and redo. As Butler explains, gender is a powerful “illusion” (Butler, 1990, pp. 185–186), created through a myriad of verbal and bodily performances. Identity is mediated by social axes, such as “race”/ethnicity, social class, and gender (Archer & Francis, 2007; Calabrese Barton & Brickhouse, 2006), which shape the extent to which particular choices and aspirations are perceived as possible and desirable (“for me”).

From this perspective, “race”/ethnicity is seen as a “fiction,” albeit one with very real, material consequences (Hall, 1990). Ethnic boundaries are shifting and contested, such that ethnic groups might be better understood as collectivities that are forever “becoming”—they are, in Anderson's (1992, p. 6) terminology, “imagined communities” organized around notions of common origins, but inflected by gender and social class (Anthias & Yuval‐Davis, 1992). Such a position rejects any notion of identity as essentialized or fixed, and treats identity instead as socially and historically located articulations of complex and shifting subject positions (Anthias, 2001).

We also draw on the concept of “science identity,” as developed by Carlone and Johnson (2007) to refer to both an individual's sense of self and the extent to which a student sees (or as we would understand it, *performs*) him/herself as interested in and/or competent at science and the extent to which a student is recognized (as being talented/having potential in science) by others (which we would interpret as referring to the congruence, or not, between a student's own identity performances and the discursive contexts within which these performances are enacted).

Our approach foregrounds intersectionality, that is, the relationship between social axes of race/ethnicity, gender, and social class. In line with intersectionality theory (e.g., Collins, 1990, 1999), we treat these social divisions as inseparable, in that the meaning of one cannot be divorced from the others. For instance, as Collins (1999) explains, this approach examines subjectivity as located within a nexus of multiple, intersecting social relations (oppressions/inequalities). Rather than an additive model (e.g., where gender might be treated as a discrete set of relations, which may be “added” to a discrete set of classed inequalities), an intersectional approach would understand the very nature of each set of social relations as interacting with, and mediated by, other social axes of inequality. Hence, there is no single “Black” identity that is not also gendered and classed—and no form of “femininity” that is not also at once racialized and classed in some way, and so on.

Our analysis draws in particular on our previous conceptualizations of the intersection of inequalities in the dominant discursive production of the “ideal student” within British educational discourse (Archer, 2008; Archer & Francis, 2007). This framework drew on empirical data to map out the ways in which the “ideal student” is repeatedly produced within dominant discourse as White, male, and middle class. This is achieved through a splitting and projection of undesirable attributes on to differently racialized, classed, and gendered others (see Table 1).

**Table 1:** The Production of the Ideal Student as White, Male and Middle Class Within Dominant UK Educational Discourse (Adapted From Archer & Francis, 2007)

“Ideal” Student	“Other” (“Pathologized”) Student	“Demonized” Student
Naturally bright Leaders Active Normal sexuality Entitled (White, male, middle class)	Attains via diligent plodding/hard work Followers/conformist Passive Repressed/oppressed Deserving poor (Chinese/Indian, female, working class)	Naturally unintelligent Rebels Aggressive Hypersexuality Undeserving (Black, White, male/female, working class)

This model seeks to explain why different ethnic groups tend to be constructed in quite different ways within education and society more generally, and how at the same time others are still constructed as problematic and different to the (often unarticulated) “norm.” For instance, students from Indian and Chinese backgrounds tend to be positioned as high achievers, as the middle column indicates, but they are still excluded from the identity of the ideal student because they are constructed as learning in the “wrong” way (e.g., as “too passive”). Black African and Caribbean students tend to be located within the right‐hand column, as low‐attaining, disruptive students who are associated with problematic (heterosexualized) masculine and feminine identities (e.g., as being “too loud,” “too sexualized,” and so on, and hence demonized (see Mirza, 1992; Sewell, 1997; Skeggs, 1997, 2004; Archer et al., 2007). As Walkerdine (1990) discusses, there is a long dominant discursive association of middle‐class femininity with “demureness” and academic achievement, against which “glamorous” working‐class femininity is Othered and positioned as “not academic.”

In this paper we focus in particular on Black students’ aspirations. We see aspirations as complex, multiple and sometimes contradictory social phenomena (Archer, Hollingworth, & Mendick, 2010) which provide a useful focus in that they are socially indicative phenomena that can illuminate the intersection of social inequalities within young people's lives. Although aspirations do not predict future outcomes, large longitudinal quantitative analyses show that aspirations can provide probabilistic indications of children's future paths (see Croll, 2008; Tai, Qi Liu, Maltese, & Fan, 2006). Evidence suggests that young people's science aspirations vary in complex ways by “race”/ethnicity and gender. For example, Riegle‐Crumb, Moore, and Ramos‐Wada (2011) found in their U.S. research that “no singular story applies to females across different racial/ethnic backgrounds” (p. 458). Their findings also showed that White and Hispanic girls are half as likely as White boys to aspire to a math career and that Black and Hispanic boys express similar aspirations to science and math careers as their White male peers, but record notably different levels of attainment. In particular, research indicates that minority ethnic students, including those who are high science achievers, struggle to identify with careers in science (Jenkins & Nelson, 2005; Wong, 2012). In this paper, we explore why Black students seem less likely to aspire to, and consequently follow, science careers.

Finally, our conceptual framework draws on the Bourdieusian concept of capital and, in particular, our more recent development of the notion of “science capital.” Bourdieu's work (e.g., Bourdieu & Passeron, 1977; Bourdieu, 1984) addresses the reproduction of social inequalities in society, proposing that relations of privilege and domination are produced through the interaction of habitus (an inner matrix of dispositions that shape how the individual operates in the social world) with capital (resources, which can be economic, cultural, social, and symbolic) and field (social contexts). In our previous work, we have proposed the notion of science capital as a conceptual tool for helping to understand uneven patterns across different social groups in children's likelihood of perceiving science careers as potentially “for me” (Archer et al., 2012a). As we discuss in greater detail elsewhere (Archer et al., 2014), we use the term “science capital” as a conceptual tool for understanding patterns in the formation and production of children's science aspirations:

Science capital is not a separate “type” of capital but rather a conceptual device for collating various types of economic, social and cultural capital that specifically relate to science—notably those which have the potential to generate use or exchange value for individuals or groups to support and enhance their attainment, engagement and/or participation in science.

Science capital thus comprises science‐related forms of social, economic, and cultural capital (e.g., science‐related qualifications, literacy, social contacts) and “the meaning and value of a particular form of science capital will vary depending on who is possessing/deploying it and in what context (field)” (ibid).

In this paper, we thus use the above theoretical toolkit to explore the extent to which Black students perceive science careers as being “for me” (or not) and the ways in which these perceptions are shaped by intersection of identities and inequalities (of material and cultural resources, i.e., capital) in relation to intersections of “race”/ethnicity, social class, and gender.

## THE STUDY

The ASPIRES project is a 5‐year study funded by the UK's Economic and Social Research Council as part of its Targeted Initiative on Science and Mathematics Education. The project explores science aspirations and engagement among 10–14‐year‐olds. It comprises a quantitative online survey of the cohort and repeat (longitudinal) interviews with a selected subsample of students and their parents, with the aim of generating both a breadth and depth of data. The survey and interviews are conducted at three time points: Phase 1 was conducted at the end of primary school (age 10/11, Year 6), Phase 2 in the second year of secondary school (age 12/13, Year 8), and Phase 3 was administered (age 13/14, Year 9). This paper discusses data from all three phases, but focuses particularly on qualitative data relating to students who self‐identified as “Black” (including those who self‐categorized as mixed race, Black/White). Brief contextual information is provided from the surveys as a means for framing the qualitative data analysis. Full details of the survey and its methods, analyses, and findings are discussed in separate publications (DeWitt et al., 2011; DeWitt, Archer, & Osborne 2013), but key information is detailed here.

The survey collected a range of demographic data (including measures of cultural capital^3^) and covered topics such as aspirations in science, attitudes towards school science, self‐concept in science, perceptions of own and others’ gender identity, images of scientists, participation in science‐related activities outside of school, parental expectations, parental school involvement, parental attitudes toward science, and peer attitudes toward school and t school science. The development and validation of the survey instrument has been described elsewhere (e.g., DeWitt, Archer, & Osborne, 2014), as have the findings from the first survey (DeWitt et al., 2013; DeWitt et al., 2014), which provides further detail on the reliability and validity of the survey instrument, as well as the specific items. The majority of questions used a Likert scale to elicit attitudinal responses.

Each of the surveys focused largely on the same key topic areas to allow for data comparison, although a few questions were added or adjusted between Phase 1 and 2, to reflect changes between primary and secondary school. Aspirations, subject choices, and post‐16 plans were also explored more deeply in Year 8 and 9 surveys, reflecting the growing maturity of participants and increasing relevance of these aspects of their lives. For instance, in the second survey, items were added about students’ perceptions of their science teachers, the perceived usefulness of studying science, and future subject choice (postcompulsory). Interview topic areas include constructions of self (in and out of school, interests, learner identity, self‐efficacy), experiences of school, experiences of and views on school science, teachers and other subjects, aspirations and the future, formation of aspirations, influences on choices, processes of decision making, imagined future subject choices, gendered constructions of self and others, extracurricular activities, images of scientists; achievement and popularity, and usefulness of science.

The first phase of the research was conducted with students when they were in Y6 (age 10/11 years). This included a nationally representative survey, completed by 9319^4^ students in England, who were recruited from 279 primary schools (248 state schools and 31 independent). This sample represented all regions of the country and was roughly proportional to the overall national distribution of schools in England as measured by attainment and proportion of students eligible for free school meals (FSM). The second phase of the survey was conducted two years later (2011/12), when participants were in Year 8 (age 12/13 years). A total of 5634^5^ Year 8 students from 69 secondary schools (58 maintained and 11 independent schools) completed the Phase 2 questionnaire between September and December 2011. The third phase was administered 1 year later, when students were in Year 9 (age 13/14 years). A total of 4600 students from 147 schools completed the questionnaire in Year 9 between February and May 2013, of whom 1043 had also completed the survey in Year 6. Again, schools in both Phase 2 and 3 represented all nine Government Office Regions in England and comprised a range of attainments at Key Stage 3 science (from 2008) and a range of FSM eligibility. In addition, the samples were roughly proportional to the overall distribution of schools in England in terms of attainment and the proportion of students eligible for FSM. Of the 4600 students who participated in Survey 3, 2038 (44.4%) were male and 2550 (55.4%) were female. (Twelve students did not provide their gender.) A total of 4438 (96.5%) attended state schools and 162 (3.5%) attended independent schools.

Students were asked to self‐categorize their ethnic background on the surveys via a double‐level question, which directed students to particular detailed choices in the second part, depending on how they answered the first, more general level part of the question. No students declined to answer this question. Students’ responses to the second level of questioning indicated their selection of one of a list of subcategories, such as White English, Scottish, Welsh, Irish, or other; Black Caribbean, African, or other. There were also separate subcategories for different mixed identities (e.g., White Black and Asian Black).^6^ Overall, Year 9 students fell into the following (self‐reported) ethnic categories: White (71.2%, *n* = 3276), Asian (13.5%, *n* = 620), Black (6.2%, *n* = 286), Chinese/East Asian (1.5%, *n* = 69), and other (7.6%, *n* = 349). This distribution among ethnic categories was similar for the Year 6 and Year 8 samples. For instance, the Year 6 sample was 74.9% White, 8.9% Asian, 6.9% Black, and 7.5% other, whereas the Year 8 sample self‐reported as 75.3% White, 10.5% Asian, 6.2% Black, and 7.3% other. Year 8 and Year 9 students were also asked whether or not they had been born in the United Kingdom and if not, how long they had lived here. Proportions were very similar between these surveys, with 4110 Year 9 students (89.3%) reporting having been born in the United Kingdom, and 490 (10.7%) had not. No students declined to answer this question. Of the students who had not been born in the United Kingdom, 49 had been here less than 1 year, 60 for 1–3 years, and 381 for more than 3 years.

In the first phase of the qualitative research, 170 interviews were conducted with 78 parents and 92 children age 10/11 (Year 6). Participants were drawn from 11 schools in England (nine states and two independent schools). Potential schools were purposively sampled from the list of 279 schools who responded to the Phase 1 survey as part of the wider study (see also DeWitt et al., 2014) to represent a range of geographic and social/economic contexts, including multiethnic urban, suburban, and rural schools. Schools were provided with information letters about the project and consent forms to distribute to all parents in the year group. All those who agreed to take part were interviewed. Students came from a broad range of socioeconomic classes and ethnic backgrounds. For Phase 2, we managed to follow‐up 85 of these pupils when they were in Year 8 (37 boys and 48 girls) and in Phase 3 (Year 9), we reinterviewed 83 students and 74 parents. These students now attended 41 secondary schools in nine areas of England and Wales.

This paper discusses interview data from all the Black (and mixed Black/White) students (*n* = 10) and their participating parents (*n* = 6) from the qualitative sample. Table 2 provides an overview of the Black student and parents interviewees (and data collected from them).

**Table 2:** Overview of Black Student and Parent Interviewees

Student/Interview Data Collected	Parent/Interview Data Collected	Ethnic Origin	Gender	SES	Main Aspirations Y6–Y9
Alan (three interviews, Y6, Y8, Y9)	Tasha (one interview, Y9)	Mixed Black Caribbean/White	Male	Low	Computer games designer (Y6/Y8) “keeping options open” (Y9)
Ali (three interviews, Y6, Y8, Y9)	–	Black African (Somalian)		Low	Engineer (Y6–Y8), business (Y9)
Cristiano (three interviews, Y6, Y8, Y9)	John (two interviews, Y6, Y9)	Black African (Nigerian)	Male	Low	Actor, statistics, doctor or lawyer
Gemma (three interviews, Y6, Y8, Y9)	V (two interviews, Y6, Y9)	Black other (Seychelles)	Female	Mid/low	Psychology/actress, business, fashion
Jake (three interviews, Y6, Y8, Y9)	Bunmi (two interviews, Y6, Y9)	Black African	Male	Low	Law (Y6–Y9)
Kelsey (three interviews, Y6, Y8, Y9)	John (two interviews, Y6, Y9)	Black African (Nigerian)	Female	Low	Teacher (Y6–Y8), business
Pamela (three interviews, Y6, Y8, Y9)	–	Black Caribbean	Female	Mid/low	Dance teacher, teacher, baker
Tom2 (three interviews, Y6, Y8, Y9)	Saadiah (two interviews, Y6, Y9)	Black African (Somalian)	Male	Low	Footballer, entrepreneur (Y8–Y9)
Vanessa (three interviews, Y6, Y8, Y9)	Robbie (father) and Akimi (mother) (two interviews, Y6, Y9)	Black African (Nigerian)	Female	Low	Doctor (Y6)
Selena (three interviews, Y6, Y8, Y9)	Saadiah (two interviews, Y6, Y9)	Black African (Somalian)	Female	Low	Nurse/police, forensic scientist

Interviews lasted between 30 minutes and 1 hour (for students) and up to 1.5 hours (with parents). Among the subsample of Black students and parents, interviews were conducted by the second author (a White, middle‐class American woman) and a PhD researcher, Billy Wong, a British‐Chinese man, with the majority of interviews being conducted by Jennifer. Interviewees were invited to choose their own pseudonyms, hence the majority of pseudonyms cited in this paper reflect the personal choices of interviewees.

Interview topic areas broadly mirrored the survey areas to explore students’ meanings, understandings, experiences, and identities in more depth. Interviewers probed responses to encourage participants to explain their views and to reflect on the potential sources or influences on their views. Brief field notes were taken after each interview. A complete copy of the survey and/or interview questions is available from the authors.

All interviews were digitally audio‐recorded and transcribed. In line with the study's conceptual approach outlined previously, data were analyzed using a discourse analytic approach (Burman & Parker, 1993) that is informed by feminist poststructuralism. As Alldred and Burman (2005) discuss, feminist poststructuralism differs from more general approaches to discourse analysis (Wilkinson & Kitzinger, 1995) in that it does not attempt a close, “micro” textual analysis but rather focuses on looking for patterned talk (discourses) within the data. This approach also pays close attention to the organization of power within the talk and the social implications of particular constructions (e.g., examining what talk is “doing” within participants’ discourse). Data were collated into “student” and “parent” groupings, in which all interviews pertaining to a particular student or parent were grouped together to enable a longitudinal “picture” of the individual over time. A summary grid was produced for each individual, detailing their demographics and responses to key questions/areas of interest over the three time points (e.g., “aspirations, Y6–Y9”). Using the grid as a guide, and moving between the grid and the original transcripts, data were initially coded by at least two researchers and organized using the NVivo software package into key themes and responses to questions. The lead author then searched the coded extracts to assign student aspirations into one of four main categories: “science aspirations,” “science‐related aspirations,” “dropped science aspirations,” and “never held science aspirations.” These categorizations were checked by the second author in line with her reading of the data. Moving between the thematically coded data and the individual participant grids, the lead author then sought to identify patterns within participants’ talk relating to constructions of science, scientists, and science careers. Data were also subjected to a theoretically informed analysis (in line with the stated conceptual framework) to identify racialized, gendered, and classed identity performances, which were then interrogated to identify any relationships between these performances, student aspirations, and student constructions of science, scientists, and science careers. For instance, initial coding was used to collate participants’ constructions of science and scientists. Subsets of constructions (which fell together as particular patterns of meaning) within these were then identified (e.g., “scientists as White men,” “scientists as brainy”). Potential relationships were tested and refined through successive phases of coding and analysis, iteratively testing out emergent themes across the data set to establish “strength” and prevalence (Miles & Huberman, 1994), which were then mapped back against participant status (student/parents), gender, and social class, to explore any potential relationships. A discourse analytic lens was applied to these subset groupings to explore the potential intersections of power (to expose the influence of classed, gendered, and racialized inequalities within participants’ constructions)—for instance, to explore the role on inequalities in relation to notions of “cleverness” and in terms of the possession of resources relating to science (“science capital”). A feminist poststructuralist lens was then applied across the coded data extracts and subsets to explore how and where particular versions of (classed, racialized) femininity and masculinity were being reproduced, asserted, or resisted (e.g., the resistance of science identity through “glamorous” working‐class Black heterofeminity). These were then retested across the sample of participants to establish prevalence and to map patterns in relation to the expression of science aspirations.

## SURVEY DATA: ETHNICITY AND SCIENCE ASPIRATIONS

The Y6 survey showed that Black and Asian students expressed stronger science aspirations than White students, as reflected by their mean scores on a composite variable composed of items related to aspirations in science (i.e., “I would like to study more science in the future,” “I would like to have a job that uses science,” “I would like to work in science,” “I would like to become a scientist,” and “I think I could become a good scientist one day”). For this variable, White students had a mean of 13.3, whereas the mean for Asian students was 15.8 and for Black students was 14.4. The difference among these groups was significant, *F*(5, 9313) = 41.98, *p* < .001, and post hoc comparisons (Bonferroni) reflected that the score of Black students was significantly lower than that of Asian students but significantly higher than that of White students.

Similar patterns emerged in the data from the Year 8 and Year 9 surveys as well. In particular, in Year 8, the means for aspirations in science were Black 14.75, White 14.03, and Asian 15.91, and in Year 9, the means were Black 15.51, White 13.81, and Asian 15.96. Although analysis of variance analyses reflected significant differences among the groups in both years (*p* < .001 for each), post hoc comparisons highlighted that in Year 8 only Asians were significantly higher than other groups: Black and White students expressed science aspirations at a similar level to one another. In Year 9, Black and Asian and students of “other” ethnicities all expressed significantly higher science aspirations than White students. However, multilevel modeling analyses (which take into account a variety of background variables, such as gender and social class, as well as other composite variables, such as attitudes to school science) suggest that although these differences exist among the groups, ethnicity does not have as close of a relationship with aspirations as other factors (such as gender, attitudes to school science, or parental attitudes to science). Nevertheless, taken together, the three surveys suggest an overall picture in which Asian students consistently have stronger aspirations in science than White students. Black students also tend to have stronger aspirations than White students, though not as strong as Asian students, although the effect sizes of these differences are very small (ω = .1). Nevertheless, as we know from participation figures, Black students’ relatively high likelihood of expressing science aspirations does not translate into later participation rates and we interpret our survey data as suggesting that the “problem” of Black students’ lower post‐16 science participation does not appear to be due to any early lack of aspiration (a finding which contradicts the assumptions in various UK government policy texts which foregrounds “poverty of aspiration” as an explanatory factor for minority ethnic underachievement and participation, e.g., Department for Education and Skills [DfES], 2005). However, it should also be noted that statistical modeling of the quantitative data suggests that, as a factor, ethnicity does not have as close a relationship to aspirations as other factors. That is, there may be some powerful common factors that are shared across groups of students, but which may be enacted in particular ways such that Black students are disproportionately likely to be excluded, or Othered, from participation in science. We now turn to the qualitative data to see if a more in‐depth understanding of the views and aspirations of Black students and parents in our sample can advance our understandings further.

## QUALITATIVE DATA: BLACK STUDENTS’ ASPIRATIONS AT YEAR 9

Analysis of student interviews from Year 6 to Year 9 (see Table 3) suggests that overall members of our qualitative sample were more likely to express science aspirations than their counterparts in the nationally representative survey. That is, and as might be expected, families (from all ethnic backgrounds) with children who held science or STEM aspirations disproportionately volunteered to be interviewed. Asian students are among those most likely to express aspirations for careers in, but particularly from, science, with 8 of 11 students expressing such aspirations in Year 9 (notably for medicine). Many of these students had consistently held these aspirations since they were first interviewed, age 10/11. Only one South Asian student had never held a science/related aspiration and two had at some point previously expressed science aspirations but had since dropped them. White students were equally like to express science/related aspirations as not, with 26 aspiring to careers in/from science, 20 never having held science aspirations and 9 who had dropped them. Likewise, almost half (4/9) of Black students aspired to science or science‐related aspirations at age 14, although it was also notable that Black students were proportionally more likely than White or South Asian students to have “never” held a science‐ or STEM‐related aspiration (5/9, or 55% as compared to 36% of White and 9% of South Asian students).

**Table 3:** White British, South Asian, and Black Students’ Science Aspirations in Year 9 (age 13/14)

	Science Aspirations (*n* = 14)	Science‐Related Aspirations (*n* = 24)	Dropped Science Aspirations (*n* = 14)	Never Held Science Aspirations (*n* = 30)	Total
White British	11	15	9	20	55
South Asian	1	7	2	1	11
Black	2	2	1	5	9

Of course, the interview numbers are small and should be treated with care, yet we still believe that both data sets have some valuable insights to offer—not least, as we discuss here, some insights into why the *majority* of Black young people (both on the survey and in the interview sample) do *not* actually aspire to science‐ or STEM‐related careers. We now move on to consider some of the factors that might influence these differential patterns of aspiration.

## NOT LOW INTEREST, LOW ATTAINMENT, OR A POVERTY OF ASPIRATIONS

Some previous literature and education policy has suggested that the reasons why Black students tend to be underrepresented in higher education, and particularly in fields such as science, can be attributed to their lower attainment, interest, and/or aspirations. For instance, a report by the Royal Society (1998) highlights attainment differentials as a key reason for minority ethnic groups’ underrepresentation in STEM subjects at degree level and UK government education policy has consistently associated underrepresentation and lower levels of attainment among minority ethnic students as the result of “a poverty of aspirations” (e.g., DfES, 2005). There are also a wealth of initiatives aimed at encouraging a greater diversity of young people into STEM, which are based on attempts to increase young people's interest in science.^7^ Yet none of these explanations were borne out by our data. For instance, only Tom2 was in lower school sets; the other students were in top sets or middle sets, with several reporting achieving high grades in their courses and being eligible to take the prestigious Triple Science option for GCSE (the highest status Science option that can be taken in national examinations age 16).^8^ As Jake said, “my teacher's telling me to go for Science cos I'm really good at it.” As Jake's mother, Bunmi, explained, this achievement was not new or recent:

[Since] primary school he's doing well in Science—he knows Science very well. Even there he knows Science very well. When they give me the report, he's very good in Science. (Bunmi, Jake's mother, Y9 interview)

There was also no evidence of low family aspirations or support in our sample. All families aspired highly for their children, irrespective of social class (two families were lower middle class and seven were working class). All parents and children described strong parental support, pushing, and high expectations for children to continue into higher education and achieve a professional career. As John (Kelsey and Cristiano's father) explained,

I am satisfied, but still I still want them to do better … I still want them to do better. Just like … if you score 70%, that's a good mark. But still you want to score 80 or 90, that's all.

John felt he was typical of Nigerian families, as “they rate education very highly” and saw high expectations as also common to Asian families (“seem as if the parents of Black and Asian—they want more from their children”). Likewise Robbie, another Nigerian father, explained,

I mean I keep saying it … it's a very difficult world out there—you don't need anybody to tell you that. So the best you can give to yourself … it doesn't matter what the economy times are saying—education, you just need to have that. And the minimum you can get in today's world is just a degree—the minimum.

Again, these findings are borne out by other studies of minority ethnic students and their families in the United Kingdom (e.g., Archer, 2003; Archer et al., 2010), and they reinforce the observations of family support for Black students to continue with science that has been noted in other studies (e.g., Russell & Atwater, 2005).

From Year 6 to Year 9, the Black families in our study reported motivating their children frequently, almost daily, to work hard and aspire to higher education. Several parents also provided practical support, such as paying for private tutoring. For instance, Tasha (Alan's mother) had already researched computer science degree courses for her son. Similarly, and echoing Lareau's (2003) conceptualization of “concerted cultivation,” Gemma's mother, V, described her parenting approach as “I will say hands on, because I want her really to do well and I want to give her the encouragement because she is doing well, yeah.” However, unlike the middle‐class parents in Lareau's sample, the Black families in our qualitative sample tended to lack the economic, cultural, and social capital of some of their comparatively more privileged White and Asian middle‐class counterparts, so their support was often restricted to providing moral support, encouragement, guidance, and motivation, rather than subject expertise, social contacts, or expensive extra curricular activities. The important role played by some African American families in the United States in motivating their children to continue studying science is also noted by Hanson (2007).

There was no evidence of Black students lacking interest in science, with all students reporting liking science to some degree. Tom2, Gemma, Ali, and Selena described themselves as “really interested” in science and named it among their favorite subjects. Alan, Pamela, Kelsey, Jake, and Cristiano described more of a moderate, or differentiated interest, either not liking science as much as other subjects or liking some aspects of science but not others.

It's basically all of it I love, I don't mind any of it. (Tom2)

Alan also talked of wanting to “take as much as I can” of science when it comes to choosing his GCSE options. As his mum Tasha proudly reflected, “He excels in Maths and Science.” These findings chime with research conducted in the United States, which shows that minority ethnic students, but particularly girls and women, tend to report strong interest in science (e.g., Brickhouse et al., 2000; Johnson, 2006). For instance, Hanson and Palmer‐Johnson (2000) and Hanson (2004) found that African American women are very interested in science, sometimes more so than White women. So if lack of interest is not the issue, what causes differential patterns in science aspirations?

In the following analysis, we explore four further possible reasons why the Black students we interviewed were proportionally less likely than Asian or White students to aspire to careers in science. This is followed by a case study of the two Black girls, Selena and Vanessa, who did aspire to a career in science to explore what may have enabled them to “think otherwise.”

### The Science = Scientist Dilemma

In line with the majority of students in the wider data set (of all ethnic backgrounds), most Black students in the interview sample were able to name only a very narrow range of potential future careers that studying science might lead to, namely as a scientist, science teacher, or doctor. Pamela and Jake's views were typical:

I think they [science subjects] don't lead to a lot of like jobs that are like around like today but I think they lead to a lot of like careers so like you could be a microbiologist or stuff like that … an astronaut. a science teacher. (Pamela, Y9)

Well like jobs that use study of science are actually important jobs, but like there's not really many there that I know of anyway. (Jake, Y9)

Doctor, scientist … That's about it. (Alan, Y9)

In this respect, the Black students and their parents are typical of the majority of participants in the wider sample, in that they expressed fairly narrow views of the types of jobs that science qualifications might lead to, even though they (like Pamela) simultaneously had a vague sense that there were “lots of jobs” that might use science or that a science qualification might be useful for. Unlike the quantitative data, which suggested that Black students were more likely than White students to express science aspirations, Black students in the qualitative sample were proportionally less likely to express science or science‐related aspirations than their White or Asian peers. As detailed in Table 3, by Year 9, only two Black students, Selena and Vanessa, aspired to a career in science (as forensic scientists), and two, Cristiano and Ali, aspired to science‐related careers in medicine. Overwhelmingly, the careers of scientist and science teacher were seen as “not for me” and, in line with the wider quantitative data from all three surveys, for most of the Black students we interviewed interest in science did not translate into wanting a science career. Tom2, Gemma, Alan, Pamela, Kelsey, and Jake were very clear that they had no interest in becoming a scientist and could not imagine themselves pursuing such a career:

I'd find it boring. Wearing a white coat, walking around with glasses. I don't find that interesting, I find getting stuck in and making money is a much better job. (Tom2, Y9)

I just don't like the thought of me being a scientist. (Gemma, Y9)

Gemma's resistance to the idea of “being a scientist” was despite her doing well in the subject at school. Her mother, V, had even prompted her to consider a career in, or from, science when she realized that Gemma was attaining highly in science at school—a suggestion which Gemma refused:

Well I was telling her [to consider] doctor or like if she's good in science, do something in this area even, forensic or things like that. But she likes to do her, she's talking about designer. (V, Gemma's mother, Y9)

In his Y6 interview, Cristiano had also stated “I can't really imagine being a scientist” and in his Y9 interview reflected:

Cristiano: I wasn't really into science then
Int: Ah okay. So what's made you be more into it now?
Cristiano: I think like … cos when I realised how important science is … [for] Education and stuff, and to get a job and stuff.

As illustrated by the above extracts, the students tended to evoke traditional, stereotypical images of scientists, which, as Scantlebury (2007) argues, tend to be narrow and excluding others. Public and popular images of scientists tend to overwhelmingly represent scientists as White, middle‐class men (Scantlebury, 2007), although popular stereotypes expressed by students may also tend to be more complex than simply reproducing the traditional “mad” or “boffin” scientist stereotype (DeWitt et al., 2013).

For most of the Black students we interviewed, a view of science as leading only to careers *in* science—jobs which they personally had little interest in—meant that although they liked science as a subject, and often did well in it at school, they did not feel it was personally relevant or necessary for their future working lives. The perceived narrowness of pathways from school science into future careers seems to be an issue that affects science more than other core subjects. Indeed, we asked students about their perceptions of the usefulness of three core subjects, Science, English, and Mathematics, and it was notable that students were much more likely to see English and mathematics as useful for “most” jobs, but science as of a more limited or restricted utility. As Pamela put it,

I think it's different because English and Maths are used more widely but Science is like a thing that you … like unless you want to be a scientist, isn't as relevant to you. (Pamela, Y9)

The perceived “usefulness” of a subject was predominantly explained in relation to the self—that is, the extent to which a student could see a particular subject as potentially relevant for their own lives and aspirations. For instance, Pamela went on to explain how mathematics would be useful for her own ambition to be a pastry chef, running her own baking business:

I think Maths is like something that's used with everything … Like anything, like … so if I want to do a pastry chef, I need like the numbers like to like weigh stuff and I'd need numbers to see how much I'd spent and how much profit I'd made.

Only Selena and Cristiano (who expressed aspirations in and from science, respectively) talked explicitly about the value of science more broadly for scientific literacy rather than just for jobs:

Cos like science is like … you need a lot … like even for humans, like if there was no science there would be no humans… You know like trees yeah, how they give us oxygen like … if people didn't know that yeah, they'll be just cutting trees and then humans start dying and that. (Selena, Y9)

Science like comes in everyday life when you're buying something and like it just gives you a little bit more information for when you're like going to the shops and you need to know where it's come from and stuff and you need to know what you need to look out for and stuff. (Cristiano, Y9)

Cristiano also gave extended examples in each of his interviews, explaining how he sees science as a key part of everyday life, including the example of a chef who dissolves ingredients, cleaners who use friction, and stunt men as exemplifying resistance and thrust.

Vanessa was slightly more ambivalent in her Y6 interview, saying “science doesn't help you quite a lot in life, but it does help a tiny bit so I do try to make an even chance of learning each thing.” But by Year 9, she also recognized the importance of science for everyday life and saw it as useful for everyday life and careers both in and beyond science.

The implications of these views, of science as predominantly only leading to jobs in science, is underlined by data from the wider student sample, which suggests that young people who see science qualifications as transferable are (i) more likely to have close family members with science degrees and/or who worked in science‐related fields and (ii) are more likely to plan to study science post‐16 and/or aspire to careers in or from science. Some Black parents recognized that they personally lacked knowledge and awareness of where science can lead, and felt that this might be a disadvantage for themselves and their children:

The problem is the lack of knowledge, the lack of awareness, where you know certain subjects like this can take them [children]. (Tasha, Alan's mother)

For example, Jake's mother, Bunmi, felt that she knew science could lead to a job as a scientist but did not actually have much of an idea what being a scientist involves, saying “I don't know the jobs they do anyway when they're a scientist.” Bunmi wanted more career advice for children like her son:

Some people they are very good in science, but they don't know which course to do … You know he [Jake] loves science—[but] what can I do? … if you do Science, what can you be later in the day? (Bunmi, Jake's mother)

Cristiano also reflected in his Y9 interview that the government should “Like introduce more jobs that include science. Cos like no one really … like understands like what job you can get like if you're going to take a subject.”

### Science and Scientists as “Brainy”: Racialized Discourses Around “Cleverness”

Irrespective of their personal levels of attainment, interest, and aspirations in science, Black students in the interview sample tended to construct science as a “hard” and “difficult” subject:

I think it [science]'s like quite hard to like understand and like you have to remember all the theories and stuff like that and like your dates and things… like there's like more to remember and I‘m like not very like good at remembering a lot of stuff. (Pamela, Y9)

Physics, I still don't like though … it just seems too complicated and I just don't get it. (Kelsey, Y9)

sometimes Science can be difficult because you don't really know what the answer is for sure … sometimes when you're doing Science like you don't really understand what you're getting taught because when you're doing Science, they have like lots of words that you don't understand. (Cristiano, Y9)

In this respect, they expressed similar views to the wider sample, in which approximately 80% of students on the Year 6, 8, and 9 surveys agreed that “scientists are brainy.” However, as we discuss shortly, in the interviews, Black students were more likely than students from other ethnic backgrounds to agree that someone needs to be clever to be really into science and were less likely to describe scientists as “normal” people (see DeWitt et al., 2013).

Black students commonly linked science careers and “being really into science” with being “brainy” or “clever.” For instance, Jake reflected in his Y9 interview that being a scientist would be “very hard.” His mother, Bunmi, also wondered whether Jake had dropped his science aspirations because “I don't know, maybe he believes Science is too difficult, I don't know.”

Vanessa's father, Robbie also concurred:

One thing about Science is—you need to be very very good. Yeah? It's almost as good as nothing having just an additional Science GCSE—you need to be able to do the triple Sciences if you really want to be … You know to carry on big time in Science. That's where I don't know whether her ability … We have let's say 120 Year 10 students, and we don't expect more than 30 or less to do triple Science. So it's not that easy, it's not very very easy.

The students tended to describe those who are really into science as “really, really smart.” (Kelsey, Y9)

I think it's the clever people (Vanessa Y9)

I think the clever people, [are into science] … to be good at Science … you kind of do need to be clever really. (Cristiano, Y9)

When Cristiano was asked by the interviewer whether he personally would ever consider a career in science, he replied that he “wouldn't mind … cos it's a good job, and it would mean I'm smart.” But Cristiano went on to explain that he could not actually imagine himself becoming a scientist because he did not consider himself one of the “smartest” in the class:

Cos … there's many people in my Science class who are smarter than me. People think like science, you need to like be really really smart like. Cos some people—they like Science and they're good at it, but they don't think they're good enough like to pursue it.

Despite attaining reasonably well (e.g., reporting attaining A and B grades) and being in the top and middle sets at schools, Cristiano, still felt that he was not exceptional enough to contemplate a career in science, saying “I'm not that good at science but I'm all right.”

This feeling of not being “clever” enough to pursue science further was also voiced by Gemma, who was in the top set for science and was attaining good grades (levels):

Like only clever people do Triple [Science] … I'm in the top set for Science but I'll still love to do Double Science, [but] I wouldn't love to do Triple, no. […] I'm a high level 6, low level 7 in Science and in top set. (Gemma, Y9)

Gemma continued, explaining how only “clever” people are into science—and that these students tend to also be unfashionable:

Well, clever people really [are into science], like people … yeah, just clever people that are really into Science … They're really good in class, like always sucking up to the teacher. Like their uniform is perfect, their ties are like really little knots, their skirts are like below their knees or they've got really like pointy shoes and like that's … yeah.

Notably, this view was somewhat more stereotypical than, and less commonly expressed by, students from other ethnic backgrounds in the wider sample (DeWitt et al., 2013). Our theoretical lens leads us to see “cleverness” as a racialized, gendered, and classed discourse, such that the identity of the “ideal” or “clever” student is not equally open to all students as a viable and authentic identity. Black students have been socially and historically constructed as “bad” students, with Blackness being aligned with intellectual inferiority within dominant racist discourse (e.g., Mama, 1995). As Bourdieu (1996/1996) discusses, the working classes are also positioned by the middle classes as “stupid,” and feminists have drawn attention to the alignment of masculinity with “natural, effortless” intelligence and femininity as attaining only though “plodding diligence” (Carlone, 2004). Hence, we suggest that the Black students’ discourse can be read as revealing traces of their positioning at the nexus of these intersecting inequalities of ethnicity and social class, with the upshot being that they find it difficult to authentically inhabit the identity of the “clever science student” due to being positioned as other in classed and racialized ways. Indeed, as noted in research conducted with minority ethnic middle‐class young people and parents, Archer (2011, 2012) found that the dominant elision of “Blackness” with “working‐classness” is such that minority ethnic middle‐class participants found it difficult to inhabit (and articulate) a “middle‐class Black” identity with any authenticity. To use Butler's (1990) phrasing, for Black students in particular, cleverness is often an “impossible” identity due to the intersection of ethnic and classed inequalities.

The difficulties the Black students experienced in aligning themselves with the “clever” identity demanded by an aspiration for a career in science was also exacerbated by perceptions of “clever” students as geeky and unfashionable. Gemma constructed her own identity in opposition to the “clever” students in her class, whom she described as unfashionable and “swotty.” Kelsey and Cristano also agreed that the students who are really into science are somewhat “geeky” and tend to be less popular than other students:

Like no one … like no one really talks to them like some people don't know them… they're like … like … like just like go around and talk and stuff, they just like keep themselves to themselves. (Kelsey, Y9)

I don't know anybody in my Science class that's popular, but it could be possible. (Cristiano, Y9)

We suggest that these constructions also reveal an intersection with gendered discourses of masculinity and femininity—such that “geeky” may be resisted as undesirable in terms of performances of popular Black heteromasculinity and femininity, and its intersection with racialized notions of Black “cool” (e.g., Majors & Billson, 1992). Views of science as “hard” and associated with clever and geeky students were also common among Black parents:

When it comes to Chemistry and Physics … I mean just the words chemistry and physics sounds difficult [laughs]. And I think with jobs that involve chemistry and physics, it's a lot of underground work, background scientific work—lab work … not a lot of people I find enjoy that […] You know people have a stereotype … well I can only say it from my perception and the people that I spoke to … you know they're quite introverted type of people who like to do that. Probably not great with outside social things. You know they're happy to be in a lab all day and work on chemicals and work out long sums and stuff. (Tasha, Alan's mother)

Brickhouse and Potter (2001) “describe some of the difficulties students face who aspire to scientific or technological competence yet do not desire to take on aspects of the identities associated with membership in school science communities” (p. 965). The dilemmas faced by the Black students in our study may also be exacerbated in this respect by the narrow, culturally limited, images of scientists discussed in the preceding section, which, as other researchers have pointed out, “don't look like me” (Scantlebury, 2007).

### Science and Science Careers as White and Masculine

As we have found in relation to the wider sample's Y6 and Y8 interviews, students and parents generally tend to associate science careers with masculinity (Archer et al., 2012b, 2013b). This association was also notable among Black students and parents. Several of the Black parents expressed more general gender stereotypical views, such as describing boys as being naturally more interested in science than girls. As Kelsey and Cristiano's father, John, put it “Seems like boys are more interested [in science], I don't know why yeah. Seems like boys are more interested.” Saadiah also said that she felt that boys are more interested in science than girls, despite evidence to the contrary from her own children, Selena and Tom2. Likewise, Gemma's mother, V, explained that she thinks boys tend to be more interested in science “because most scientists are men.” And Vanessa's father, Robbie explained,

I think the females also see science as … something you need to think about … or something you need to have your hands on. And the females are more comfortable with listening and wanting to write down… So they see it more as a man thing. …If a male student is playing with electrical power packs, the female student will have a different approach … it's just natural.

It was also notable that boys tended to be more likely to be constructed as “clever” by parents, even if their sons were not currently attaining as highly as daughters. For instance, Saadiah talked about how her son, Tom2, is doing very well at school and is “cleverer” than his twin sister, Selena, even though he has had to change schools due to behavior problems and is currently in lower sets and is attaining less highly than Selena. These gendered views are not restricted to Black parents but have also been found to be common generally in society and have been noted among teachers, including science teachers. For instance, Carlone (2004) found that boys in an advanced physics class tend to be constructed by teachers as “naturally” good at physics, even when they are attaining less well than girls. Drawing on dominant gendered discourses, girls’ higher attainment was explained as being the result of their plodding diligence, whereas the boys’ “underattainment” was rationalized as the result of their adolescent “laziness.”

Although students’ views on gender appeared to be less stereotypical than their parents, there was a suggestion that gendered views in which science is associated with masculinity might intensify with age. For example, in her Year 6 interview, when asked whether she thought there were any differences between girls and boys in terms of how much they like science, or not, Pamela answered,

Not really. Actually more girls like science than boys [Int: Why?] uh cos I think a lot of boys think that you can't be cool if you like science. [Int: Really?] Definitely a lot of boys in my class [think] um, you can't be like popular if you like science. [Int: and how do you know they think that?] Uh, cos they say it. Cos like boys on my table, they say it, and boys on like everyone else's table say it as well. [Int: So what do they say?] That science is weird and stuff. […] They don't like … they don't want to do it because they think they can't be popular if they like it. (Pamela, Y6)

But by Year 9, Pamela reflected “I think in our school mostly it's boys that are into Science.” It may be that as they get older, young people are more likely to reproduce popular societal stereotypes around gender and science.

As we have discussed elsewhere in relation to the Year 6–8 interview data, “girly” girls are generally less likely to express science aspirations than other girls, and are more likely to drop aspirations for careers in science as they get older (Archer et al., 2012, 2013). In light of this, it was perhaps unsurprising that the more “girly” Black girls, like Gemma, had never expressed science aspirations, whereas Selena, who did aspire to a career in science and who is discussed further in the final section of the paper, performed a more “tomboy” gender identity. Selena's performances of femininity from Y6 to Y9 were, in her own words, not girly but “tomboy,” which, when combined with her performance of “clever” student identity, rendered science aspirations both desirable and more congruent with her identity performances. In contrast, Gemma describes herself in her Y9 interview as into fashion: “Girly stuff but also sporty stuff like you can get these shoes that are high heels but they're like trainers but high tops, I like them.” Drawing on an intersection of gender and class, through the notion of “glamour” (Skeggs, 1997), Gemma's mother, V, concurs “Like she [Gemma] said [she aspires to] designer or hairdressing. You know all this glamour thing yeah… she likes her nails, her hair, drawing, you know.” As exemplified by the discursive construction of other students, outlined earlier in our theoretical framework, V's talk hints at the discursive association of middle‐class femininity with demureness and academic achievement—which Gemma might be interpreted as resisting through her performance of “glamorous” working‐class femininity. We suggest that these discursive associations may be amplified in the case of science (which is dominantly constructed as White, male, and middle class) such that identifying with science may be particularly challenging (and require considerable identity work) for “glamorous” working‐class Black girls. As noted in a number of U.S. studies, girls tend to be less likely to identify with science, and this negatively impacts their progress and participation in postcompulsory science and science careers (Brickhouse et al., 2000; Brickhouse & Potter, 2001; Calabrese Barton et al., 2008; Carlone, 2004). As Gemma discussed above, her femininity was also constructed as “not brainy/swotty”; thus, for Gemma, popular associations of science and science careers with both masculinity and “cleverness” render such aspirations undesirable and incompatible in identity terms with her own production of self. Moreover, the dominant construction of science as aligned with *White* masculinity renders it even more “unthinkable” in relation to Black femininity.

This association of science and science careers with White (middle‐class) masculinity is also potentially problematic for Black boys, who are often demonized within popular discourse and excluded from dominant constructions of “normal” masculinity. Numerous research studies have drawn attention to the ways in which Black masculinity is dominantly constructed as a “risky” and “dangerous” educational identity (e.g., Sewell, 1997) and the popular association of Black masculinity and low attainment, as discussed above, also contributed to rendering science aspirations less thinkable for Black boys in the study. In other words, popular associations of science and science careers with a particular version of masculinity (White and middle class) work to exclude *both* Black boys and girls from seeing science as a normal, conceivable, and achievable potential aspiration. The “risky” nature of Black masculinity (which is amplified through its intersection with working‐classness) was also evident in a number of parents’ concerns about their sons, commonly expressed as worries about peer groups. For example, Bunmi made a couple of references to her concerns about who Jake is “mingling” with in and outside school, adding “because he's a boy, and you know how boys are in London.” Saadiah also expressed concerns about Tom2's behavior (“Yeah he was naughty every time … and he was rude in the class, sometimes naughty. So they sent him over to another school”) and Alan's mother, Tasha, described her relief and pleasure that her son now appeared to be mixing with a new set of friends from more “respectable” (Skeggs, 1997, 2004) social backgrounds, rather than the “troublesome” boys from their estate:

A lot of the parents [at his new school], you know they work hard, most of them are financially stable, plus they're educated … so it's nice that [Alan] is kind of with those type of kids, cos it's not about being on the street and you know “I want to look like Fifty Cent” … it's none of that, they're just normal kids doing normal things. … He's not part of the hoody culture, as you know people like to label these teenagers and stuff. I don't want him to be part of that, I want him … it's nice because … okay we've lived on estates and we've been brought up on estates, so he sees both worlds. So it's like he's streetwise, but you know he's not street.

### Inequalities in the Social Distribution of Science Capital

He's very good in Science, I don't know why he wants to be an accountant. (Bunmi, Jake's mother)

Our analysis noted a relationship between a family's possession of science capital and the likelihood of child expressing science aspirations. Generally, this relationship appeared to hold true for the Black families in the interview sample, with low levels of science capital corresponding to low expression of science aspirations.

The Black families in our sample tended to possess little science capital. For instance, Saadiah explained bluntly “I don't know about the science” and Bunmi admitted “I don't like it [science].” Despite her nursing background, V, Gemma's mum, still found science hard and uninteresting:

I will say I'm not interested in science, but like in nursing you have to have some (inaudible), because everything we do in nursing you have to have some bit of science beside it, so I don't know really … I see science as difficult.

In this respect, they exhibited some similarity to the small number of urban underprivileged mothers in Carlone et al.'s (2011) study who regarded science as an “untouchable domain” and whose accounts were characterized by views of science as “hard” and disliked. As Carlone et al. argued,

Low levels of participation and achievement in science by underprivileged urban children are often exacerbated by the role their parents have in education. These parents are typically not involved in their children's education at the level expected by their children's teachers. Although in contemporary popular culture discourse the parents receive full blame for this lack of participation, sociological studies in education have shown us that this is not a fair assessment. (Carlone et al., 2001, p. 692)

Out of all the Black students in our study, only the four with science/related aspirations reported doing any science‐related activities in their spare time. For example, in her Y6 interview, Selena described replicating school experiments at home. She also looked up science quizzes and information on the computer and watched science‐related television programs. She also recounted considerable science interest among her siblings, saying of her twin brother, Tom2, “he always talks about science” and her older sister “She loves things about science … cos she's got lots of books about science.” Vanessa also talked about using science kits and resources (e.g., her microscope) provided by her father.

From Y6 to Y9, Cristiano and Alan both appeared to tinker a lot at home: Cristiano explained how he did little “kitchen science” experiments at home, played science games on the computer and watched science TV programs. He described how he invented new contraptions, such as putting together two containers to make a new measuring tool for cooking, and also talked about fixing computers and tinkering with mechanical things. Alan had a long‐standing obsession with tinkering with technology, something that his mother, Tasha, talks about at length in her interviews:

Alan is interested in tinkering and has tried to take apart computer at home to see how it works.

Tasha describes how, through his tinkering, Alan has become very adept with technology, even to the point of being banned by one of the games companies for hacking:

Yeah they banned him. So he's able to do all these things, and I don't know how he's doing them … No one's taught him—he's taught hisself, and that's what's amazing about him. I know some of the stuff he's not supposed to do, but the fact that he knows how to do it and no one's showed him, it's beyond me.

That those children who reported doing more STEM‐related activities in their leisure time were also those who expressed STEM aspirations suggests the potential recursive relationship between behaviors, interest, and aspirations. Interest may prompt children to be more likely to want to engage in particular types of leisure activity, but their exposure and practice of particular science‐related behaviors may also reinforce and prompt further interest, not least due to the development of particular competencies. As Azevedo (2011) argues, hobbies and regular leisure activities are important for the reinforcement and development of particular lines of interest. Yet, it was also notable that, apart from Vanessa, the Black students' out‐of‐school STEM activities were very much self‐led/initiated and tended to use resources that the young people themselves had sourced or accessed. This compares starkly with the highly resourced, often adult‐organized science‐related experiences that were reported by the science aspirant, White middle‐class students in the wider study enjoyed access to, by dint of their greater familial economic, cultural and science capital (see Archer et al., 2010, 2012).

The importance of capital for facilitating young people's attainment of their aspirations was underlined by Alan's mother, Tasha. She talked eloquently and poignantly about the effects that a lack of capital, and an impoverished, difficult upbringing, had had on her own life. She was highly motivated to ensure that Alan could have a better life, and recognized his need for support:

He definitely needs support. I never had that, so I found it really difficult to try and find my place within society. I never knew what I wanted to do, I never knew what I could be … I was always confused … cos certain things wasn't nurtured in me. So I think that is really important and that's something that I've taken from my own experience to my children. (Tasha, mother of Alan)

To this end, Tasha had already begun researching potential IT degree courses and had found one in a city where Alan's aunt lived, which would enable Alan to live with them while studying to reduce costs.

Several of the Black families in the qualitative sample appeared to be disadvantaged not only by a lack of science capital, but also lower possession of other types of capital too, compared to other families in the sample. For example, they tended to be among the more economically disadvantaged families in the sample, and parents in particular talked about a lack of cultural capital that hindered their ability to navigate the education system effectively. It was notable that those families who did enjoy more cultural capital, like John's (whose family was severely economically disadvantaged but who had educational cultural capital as the result of having been a secondary school teacher in Nigeria) talked about their greater confidence in navigating the educational system and supporting their children's education.

John talked about the effects of disadvantage on his own life and his subsequent commitment to motivating and supporting his children to achieve better. He described himself as “not good at science” and recounted how he was put off science by a friend at school, but now regrets not learning science. As he explained, due to being uneducated themselves, his own parents never realized that he was not attending classes, nor thought to check:

Unfortunately my parents were not educated. They can't say “What did you do today?” Just go to school, come back home [say] “Good morning,” that's all. They don't check my record, they don't check. Whether I actually attended something.

John had trained and worked as a teacher in Nigeria, but his qualifications were not recognized in the United Kingdom and he could not afford to retrain. He now worked as a low‐paid care worker. He had a profound belief in the importance of education and paid for private tutoring for all his children in mathematics and English throughout the course of the project.

All the Black parents in the interview sample described pushing their children to achieve “better lives” through social mobility. For some, this contained echoes of children attempting to fulfill thwarted parental aspirations. As Bunmi explained about her son Jake's aspiration to become an accountant, “I don't want to discourage him … because I wanted to be an accountant as well.” But Bunmi also felt disadvantaged by a lack of cultural capital, as she did not know what subjects Jake would need to study to become an accountant, nor whether he would need to go to university. The family did not know anyone who is an accountant and Bunmi described being at a loss to understand the UK system and dependent upon the school for advice and help:

Well in this country I don't really know what are they doing … I know in my country if you want to be an accountant or banker you have to be very good in Maths and English…. I don't know about this country you know… So if he say he want to be an accountant, I think they should be tell him in school the kind of subjects, what he's got to be good, what you have to be good in.

In sum, we suggest that the disadvantaged structural location of Black families, particularly those from working‐class backgrounds, means that they are disproportionately likely to be excluded from the possession of science capital, which will negatively impact on the likelihood of children developing or sustaining science aspirations. This is potentially an issue for all working‐class families, but may be amplified by the intersection of racial and class inequalities, as experienced by many Black working‐class families.

## BLACK, FEMALE, LOW SOCIOECONOMIC STATUS … AND ASPIRING TO A SCIENCE CAREER: THE CASE OF SELENA AND VANESSA

So far we have argued that it is not unusual or unexpected that Black students in our qualitative sample tend *not* to aspire to science, because they are situated at the nexus of intersecting inequalities, which render science “unthinkable” for four main reasons: (1) Black students are structurally excluded from dominant notions and representations of the “typical scientist”; (2) they are Othered from dominant associations of science with “braininess”; (3) due to intersecting inequalities which increase the likelihood of Black families experiencing economic and social hardship, they often lack capital generally, and science capital in particular, that might enable them to see science as transferable and to navigate and strategically “play” the system; and (4) black girls and boys are Othered by popular associations of science careers with White masculinity, with girls particularly disadvantaged by their distance from these privileged identity positions, rendering science a less possible/desirable potential aspiration for Black girls.

As discussed earlier, three Black boys aspired to STEM‐related careers: Cristiano and Ali aspired to become doctors (although Ali primarily aspired to a career in business) and Alan aspired to a career in IT. Two Black African (Nigerian) girls, Selena and Vanessa, aspired to a career in science as forensic scientists. As Gramsci (1971) notes, no hegemony is ever complete—there are always spaces of resistance, transgression (Butler, 1990), and hope (Apple, 2013). Here, we focus in more depth on Selena and Vanessa, asking what makes it possible for these two Black girls to overcome the odds to aspire to careers in science?

Selena and Vanessa are both from Nigerian backgrounds and live in an economically deprived inner‐London borough. They both attend a single‐sex school and both come from low‐income/SES households, although Vanessa's father, Robbie, studied pharmaceutical science at university back in Nigeria and now works as a science technician in a local urban secondary school (a low paid job, often hourly paid). Vanessa's mother, Akimi, who was also interviewed, is a care worker. Selena's mother, Saadiah, speaks very limited English and does not work.

### Attitudes Toward Science

In their Y6 interviews, Selena and Vanessa seemed somewhat ambivalent about science:

I like kind of science but not all of science. (Selena)

I would describe myself into, um, kind of okay with science, because sometimes science is a bit boring, sometimes it's interesting. (Vanessa)

Like many other students, they described how they enjoyed doing experiments, but not writing. In Year 6, Vanessa named PE as her favorite subject, although she also liked science. She felt her interest in science to be unusual among her primary school peers, saying that most of the girls and boys she knows do not like science and find it “boring.” She related her own interest in science to her family's African origins:

But actually some African people like science a bit more, because science in Africa seems to be what's getting more money at the moment, because science people like to look of germs and sicknesses, because in Africa … they get sick a bit quickly, so they like doctors and scientists who can find out how it's happening and it's going around.

By Year 9, both Selena and Vanessa now liked science and named it as one of their favorite school subjects, with Selena expressing the strongest sentiments, saying “I just love science, [I] like the way everything is in science.”

### Aspirations

At age 10, Selena aspired to be a nurse or a police officer, although she admitted that her family was not keen on these jobs. Selena had never considered being a scientist (“I've never like thinked of being a scientist, cos it's hard”), evoking the notions of braininess and narrow representations of scientists discussed earlier. By age 14, Selena aspires to be a “forensic scientist or PE teacher … Something to do with Science.” Selena saw both of these aspirations as having some relationship to science, explaining, for instance, how PE teachers relate to science: “like when you're learning about the muscles and fitness part.”

In her Y6 interview, Vanessa aspired to be a doctor, explaining:

When I was a baby I wanted to be a singer, but now I'm starting to feel like being a doctor because I think I like helping people when they're hurt and I like looking at blood.

Vanessa explains that her medical aspirations come from her mum, who is a care worker but who had wanted to enter medicine:

But she [mum] did want to be a doctor, but she didn't get to […] They really want me to be a doctor as well.

But by age 14, like Selena, she aspired to be a forensic scientist, explaining “I don't really want to be a doctor anymore.” Instead Vanessa aspired to pursue a science career, like her father.

The two girls’ aspirations to become forensic scientists were closely linked to their TV viewing of popular crime drama programs, notably *CSI*:

cos I like watching stuff about science, I want to know how it … things that happen for forensic scientists. (Selena^9^)

Vanessa also mentioned how her ideas were formed through watching a number of programs, notably CSI NY, CSI Miami, and Death in Paradise (a British crime drama, which is set in the Caribbean and features prominent Black female and male characters). Vanessa also mentioned that her interest had been stimulated by a forensic‐style detective investigation exercise that they had done at school in Y7.

Both girls also held other aspirations alongside their interest in forensic science. For example, Vanessa was very interested in drama and during Selena's mother's home interview, Selena (who periodically entered the room and interjected) talked about also wanting to become an artist or teacher, interrupting her mother's interview at different points saying “I definitely want to be a teacher” and “I want to be an artist.”

### Academic Confidence and “Braininess”

Both girls aspire highly and are strongly supported and motivated by their parents to go to university. Of the two, Selena feels confident in her abilities and reports higher attainment. Although wider research has noted that girls in general tend to be less confident in their abilities with STEM subjects, such as physics and mathematics (Mujtaba and Reiss, 2012, 2013), high levels of academic confidence have been noticed among African American young women (Hanson, 2009), which some commentators have suggested reflects a tradition among some Black families to empower young women with self‐confidence and self‐assertion (Hanson, 2009). Both girls feel that the job of forensic scientist is achievable and think that success will depend only upon their own hard work and motivation (e.g., “If I'm serious about it, like if I don't mess about. I used to mess about a lot in secondary, but now I've changed,” Selena).

Selena differs from Vanessa in that she aligns herself unambiguously with a highly motivated academic identity, describing how she is motivated to take the “highest” subjects, for example, Triple Science:

Yeah, and I love Science. I want to get the highest one. Cos BTEC, if you do BTEC it's not a GCSE, you just get a pass and Foundation is worse as well.

Reflecting on the pathway to becoming a forensic scientist, Selena says,

Yeah, I'd need to do A Levels, go to college and uni for that. And go to a good one, because like if you go to like … don't know, like if you go to a good university yeah, you get more like … I don't know like … you get more higher things.

Selena aspires to attend not just any university but one of the “best” universities, such as Oxbridge (“Because like I think that's the best one”), and describes gaining this cultural capital from one of her teachers at school. As data from the United Kingdom (e.g., Department for Education [DFE], 2014) and United States (Calabrese Barton et al., 2008) indicate, Black (African American) girls tend to outperform Black boys academically, and this may also help engender girls’ sense of confidence in their abilities. Moreover, both attend a single‐sex school, which research shows are associated with higher rates of female participation in post‐16 science (Institute of Physics, 2012). As Mujtaba and Reiss (in press) also discuss, girls who plan to study advanced level mathematics and/or Physics also tend to be more competitive than their female peers who do not plan to study these subjects post‐16. We suggest that Selena might be interpreted as performing “brainy” identity with a degree of confidence and authenticity, which bodes well for her continued progression along a science trajectory.

In contrast, Vanessa does not inhabit a “brainy” identity. In her Y9 interview, when asked about her attainment, she says “Uh it's not amazing but it's not like rubbish so it's just middle, halfway.” She is in the second (rather than top) attainment set at school and Vanessa describes herself as “I think I'm an okay or goodish student.” Yet she too is confident that hard work will lead to success:

I think if you do work hard and like your GCSEs come out well, if you get chosen by the good college or uni then if you work hard in that too and get your A Levels, then when you get all your certificates and your qualifications then I'm sure you can actually get whatever you want to do, you can do it.

However, Vanessa later reflects that economic barriers could also prevent her from going to university (“if I don't have the money to pay for it in the future then that would probably stop me from going”). Vanessa's view of herself as a “middling” student is also reinforced and solidified by technologies of power enacted within the education system, such as the provision of differentiated careers resources:

Yeah, it depends on … I think it's a booklet and it depends on how your levels [grades] are. If your levels are really low, you get the lowest booklet, if you're middle, like alright, then you get another booklet that's a bit higher with more opportunities. And if you're exceeding, like really, really … then you get a booklet of like loads and loads of opportunities.

Vanessa went on to explain that she expected to be given the “middle” booklet, reinforcing her view of herself as a “middling” student.

### Femininity

Echoing findings from the wider study which show that “non‐girly,” high achieving girls are more likely to aspire to science careers than “girly” girls, neither of the two girls were especially “girly.” Indeed, both noted in their Year 6 interviews that they differed from other girls in their class in this respect, with Selena commenting that she is different from her female classmates because they are “like girly girly,” whereas she is a “tomboy” who loves playing football. Likewise, Vanessa complained “there's other girls in the class who are just a bit too girlie.” While Vanessa performed a moderate and restrained version of femininity, Selena performed a more explicitly “tomboy” gender identity from Y6 to Y9 interview, for example, describing herself age 14 as

I think I'm more different [to other girls]… Cos like all the girls … they're more like into other stuff, not football.

Selena retains a love of playing football, which she recognizes as “quite unusual,” although she was happy to have found some other girls at secondary school who also enjoyed playing. Her parents still do not really approve of her “unfeminine” pastime (“They said that it's not good, cos like it ain't going to get you nowhere … it's more like a boys’ thing”).

Vanessa was not as “tomboy” as Selena in her performance of femininity, but her mother, Akimi, talked repeatedly during her Y9 interview about her relief that Vanessa does not engage in excessive performances of sexualized femininity, but rather prioritizes education. Indeed, Akimi makes repeated reference to how her daughter is “natural” and does not wear make‐up, like many other girls at her school, emphasizing that Vanessa is not one of “those ones [who] they smoke or dress roughly, [and wear] the make‐up.” In this way, Akimi's talk hints at the intersection of gender and class, in which she align's her daughter's performance of femininity with more “middle‐class” (respectable, demure, and academic) femininity (Walkerdine, 1990), and against (dominant constructions of) “excessive,” “vulgar,” and hyper‐(hetero)sexualized working‐class femininity (Skeggs, 1997).

Both girls were also part of high‐attaining, “modest” female peer friendship groups. For instance, Selena describes herself and her friends as aspiring highly, attaining well, and being really into science:

Yeah they're like me, but … cos one of my friends wants to be a dentist and she likes Science a lot and that. And then she brainwashed my other friend into Science as well, so now she wants to do that as well.

Vanessa also talked about consciously sticking with academically focused friends and even having asked her teacher to move her away from disruptive friends in Science:

I try and stay away from the ones that are distracting you, the ones that want to kind of aim higher, try and stick around with them […] Yeah, me and one of my friends, she's like really smart and she really like … she's really into education, she has her like … you know, fun time and everything but when it comes to in like the class and stuff, she like concentrates. Yeah, and recently I told my Science teacher I don't want to sit near my friend so she moved me.

Her mother, Akimi, agreed, saying “she has her friends, they are nice, they are disciplined children.” Akimi's comments around “respectable” femininity evoke notions of “respectability” (Skeggs, 2004). As Walkerdine (1990) and others have written, dominant, idealized notions of the “good schoolgirl” are constructed as unsexualized, demure, and “respectable.” Working‐class and minority ethnic girls who perform more sexualized and “glamorous” forms of femininity tend to be judged as falling outside the image of the ideal student and may experience more problematic relationships with teachers and schools, as girls resist attempts to regulate their “unruly” behavior (e.g., McRobbie, 1978; Skeggs, 1997). As Black women, Selena, Vanessa, and their mothers might also be interpreted as negotiating femininity in the context of dominant racist discourses, which have traditionally aligned Black femininity with excessive sexuality (e.g., see Mama, 1995). Hence, their desire to claim an authentic, viable academic identity is facilitated by performances of a form of femininity that is coded as “intelligible” and congruent with “good student” identity within dominant discourse, that is as nonsexualized, moderate, restrained, “demure,” and “respectable.”

### Family Cultural Capital and Science Capital

Both girls benefitted from their families’ academic cultural capital, which helped parents to navigate the education system and promote their daughters’ attainment. For instance, Vanessa's father, Robbie, talked about accessing school league table information and understanding of the UK educational terminology of “levels” and “targets,” which enabled him to understand and assess his daughter's educational progress and put in place measures to help raise her performance:

She's just working averagely on that level. Nothing so like … like she's not working at a level that I would say “Wow, she has beaten my expectations”—no, she's just all within her targets… we have started a programme at the beginning of Year 9 where we have structured a home study plan for her… Every Friday she goes to a private tutor. But every other day she's got at least an hour and a half of home study where we just do work together. And I think it's been very helpful.

Both families paid for their children to receive private tutoring, a trend that has been noticed among other high attaining minority ethnic groups in the UK, such as the British Chinese, who often prioritize this support even when financial resources are tight (Archer & Francis, 2007). Vanessa's mother, Akimi, commented how having a highly educated extended family provided both practical help and contributed to children's high aspirations:

I come from a very educated home apart from me—my sisters, niece, cousin were all … All of them, they are in high position in this country. … So the lowest is me, I'm the one that is most—every other person they are really high paid people. So when she look at them, the way they live, the way they do “Momma I want to be like this”… like my dad, all my brothers and sisters are teachers, ministry workers, work with council, teachers, police, you know.

However, a key difference between the girls was their familial science capital. Vanessa's father, Robbie, was unique among the Black parents in that he possessed a high level of science capital, by virtue of having studied science at university in Nigeria and having since worked as a science technician in England. He was very “hands on” and supportive of Vanessa's science learning, although still felt that he could not be complacent:

I still feel I probably am not doing enough, but uh … you know it's all about having time and being very very determined and focussed. But yes, I have a knowledge … I mean from now to her A Levels there is hardly anything I cannot help her with.

He was also passionate about science, saying “I love everything science … [but] I am very very careful not to make it ‘because I love Science she has to love Science.’” Vanessa has long been aware of her father's love of science, and talked at length in her Y6 interview about how “my dad's into science” and how he provided her with a range of science resources. Robbie concurs in his Y9 interview that he still purchases a range of science‐related educational resources to support Vanessa's engagement with science.

Robbie admits to his own bias toward science, giving an example of how several teachers had said to him at parents evening that Vanessa is good at PE and history and should choose these subjects. But as Robbie confesses:

Now these are subjects that are not very appealing to me to be very sincere, but then again if she's good at those things I can't force her to do Science.

While he was keen not to “force” Vanessa to love science, it was notable over the 4 years of the study that Vanessa had brought her interests and aspirations more into alignment with her father's. Indeed, in Y9, the family notes that Vanessa is actually attaining better in arts and humanities subjects than in science, but this does not affect her post‐16 plans and aspirations.

In her Y9 interview, Vanessa's liking for science and confidence in the subject are clearly linked to her father's science capital. When asked to explain why science is one of her favorite lessons she says “I just like what we do, she's really nice, our lessons are fun and if I don't get it, it's like better because my dad can then just come and help so it's easier for me to learn.”

This process of gradual alignment between the aspirations of the child and the parental occupation results, as we have written elsewhere, from the interaction between family capital and family habitus (Archer et al., 2012), such that young people like Vanessa come to see their science aspirations as “natural” for “people like us.” Indeed, when asked in her Y9 interview if there was anyone who she looked up to or would like to be like in the future, Vanessa replied touchingly “My dad, he's like really good at Science and Maths, so like I would really like to be good at that.”

### “Safe” Routes? Family Preference for Medical Careers

While strongly supportive of their daughters’ higher education ambitions and their plans to study science post‐16, both families seemed to be less keen on, or slightly wary of, their daughters’ aspirations to become forensic scientists and both mothers tried to steer their daughters toward their preferred option of medicine. In her Y6 interview, Saadiah described her family as typical of other Somalian families, who want their children to become “A doctor, nurse or pharmacy or something like that.” Selena concurs in her Y9 interview, saying that her parents want her to become a doctor because “every like parent wants their daughters to be doctors and dentists.” The following extract is taken from Saadiah's interview at home, when Selena is Year 9. Selena has walked into the room and joins the conversation:

Int: Yeah yeah yeah. And what would you like to see her [Selena] do?
Saadiah: Mmm?
Selena: Scientist.
Int: Yeah? Would you like to see her be a scientist?
Saadiah: Mm … maybe doctor. [laughs]

A similar exchange occurs in Vanessa's interview, where her mother also interjected:

Vanessa: I don't really want to be a doctor anymore.
Int: Yeah, what's made you kind of go off that?
Vanessa: Because I found the forensic thing more interesting, the doctor is kind of like … and then I found the doctor thing can be a bit … could be a bit scary because if you don't save someone's life, then it's kind of your fault and you see a lot of blood and hurt people. But Forensic Science is just clues, like …
Akimi: I say let her do pharmacist.

Like Saadiah, Vanessa's mother Akimi had some concerns about her daughter pursuing a career in forensic science and would prefer her daughter to opt for a medical career instead:

It's a dangerous job, …But for me [I say] “why don't you do pharmacist? It goes on science too. You can just become a pharmacist, maybe in the hospital”. … But she said “I don't take tablets, how can I tell people to take tablets?”

Akimi also worried about her daughter's desire to emulate her father, saying “she doesn't want to disappoint the dad, that is a problem. She want to live the way the dad want her to live.”

Vanessa also explained that she tends not to talk to her parents too much about her forensic science aspiration because they try to steer her instead toward other (medical) routes:

It's just sometimes when we're watching something and it would just be like “Oh, what do you want to be?” and then I say and then they would be like “Oh, don't you want to do something else? Or this?” and I'd be like “No.”

In other words, forensic scientist appears not to be as “thinkable” an aspiration in Saadiah's or Alimi's eyes. Indeed, these mothers’ concerns may be well founded, as research indicates that Black women do face continued, considerable barriers and inequalities in pursuing science careers (Carlone & Johnson, 2007). Yet family encouragement also seems to be very important in helping Black women to continue on science trajectories.^10^ For instance, in their study of 11 successful African American degree students, Russell and Atwater (2005) found that family support was one of the several critical factors identified.

### Summary

Selena and Vanessa's cases provide an encouraging illustration of how the hegemonic impossibility of “scientist,” as a viable and desirable identity for Black students, is not absolute but can disrupted and transgressed. There appear to be several factors at work which help prompt and sustain their science aspirations. Common to both of the girls are their enjoyment of school science, their academic orientation and motivation to behave and attain well at school, their family educational cultural capital, their “nongirly” gendered performance of self, being part of academically likeminded close friendship groups, and identification with desirable images of forensic scientists in the popular media. Specific factors for each girl include Selena's identification with and performance of “clever” student identity and Vanessa's family science capital.

Although both girls seem motivated to pursue their ambitions, we also sound a note of caution, as there is a potential precariousness to their identity work. First, their structural positioning as poor, urban, Black girls suggests that they will need to work extra hard to prove themselves and maintain their aspirations. Accounts from women of color who are successful scientists highlight the ongoing difficulties and inequalities that challenge such women in their trajectories, requiring considerable resilience and support:

In reality, these women have to fight for their identities, performing, developing, and achieving them again and again in different contexts and across time. (Carlone & Johnson, 2007, p. 1208)

Second, neither is single minded in their aspirations. Other research notes that multiple and provisional or future outcome‐dependent aspirations are a common pragmatic risk management strategy among disadvantaged urban young people (Archer et al., 2010), but expressing and pursuing a single, consistent aspiration over time is more likely to lead to young people's successfully attaining that aspiration (Yates, 2008). Third, their aspirations to forensic science may be potentially risky choices. Forensic science has grown in popularity at degree level, which has led to such courses becoming competitive and oversubscribed.^11^ Research indicates students attracted to forensic science as the result of TV representations are likely to have unrealistic expectations of the course and may be at greater risk of noncompletion (e.g., Weaver, Salamonson, Koch, & Porter, 2012). There has been widespread concern about the closure of the UK public sector forensic science service in 2010,^12^ with associated uncertainty regarding job outcomes for these graduates. Moreover, because forensic science is one of the more popular, “glamorous” area of science (as portrayed by CSI‐style popular programs), it is not clear whether the girls’ science aspiration would transfer to other potential careers in or from science—i.e., does their perception of forensic scientists as potentially “like me” extend more generally to all, or other sorts of, scientists. Fourth, both mothers appear to have some concerns about their daughter's aspiration to forensic science and may not fully support this ambition. This may be particularly important given the emphasis placed by wider studies on family support as a predictor of postcompulsory science participation (e.g., Mujtaba & Reiss, in press; Hanson, 2007).

## CONCLUSION

Our study focuses on students’ science and career aspirations and in this paper we have explored in greater depth why Black students appear to be less likely to aspire to a career in science—and what makes it possible for two Black girls, Selena and Vanessa, to see a science career as being potentially “for me.” We argue that these students’ aspirations are important because they reveal the intersection of inequalities of “race”/ethnicity, gender, and social class, which makes science less “thinkable” for Black students and presents additional challenges for science‐aspirant students to overcome to maintain their aspirations over time. Our concern is not only that these inequalities impact the science “pipeline,” limiting diversity, equality, and representation within the science workforce and industry; nor is it only that ensuring an equitable spread of scientific literacy across all social groups is an important equity project. Rather, our concern is also that we view uneven patterns of aspiration as a potential social injustice in itself. That is, a socially just society should provide equitable resources, opportunities, and possibilities to enable all young people to consider a wide and full range of careers as potentially possible and “for me.” Moreover, all young people should be able to benefit from resources and support to help them to navigate their chosen career pathways. Careers in science need to be made more open and “thinkable” for all—the extent to which careers are seen as achievable and “for me” should not be limited by students’ social structural locations. The absence of Black students from prestigious science routes is a social injustice in itself that needs to be addressed in its own right. Indeed, the underrepresentation of particular social groups within science careers arguably reinforces the cycle, making it “twice as hard” for those who do attempt to follow science trajectories.

We have argued that although the “being/doing divide” (liking science, but seeing science careers as not for me) is common across all students (Archer et al., 2010), it is particularly problematic and exacerbated in the case of Black students. This is in no small part due to sociohistoric dominant discourses that align science and scientists with White, middle‐class masculinity, which prevents many Black students from seeing science careers as being “for me.” We have also attempted to show how such associations are complexly formed, being particularly entangled with the notion of “cleverness.” Our analysis indicates that science is strongly aligned with cleverness in popular discourse, but we would argue that this association may also be produced through institutional structures and technologies, which tend to result in less privileged social groups being concentrated in underresourced and lower status schools, neighborhoods, and qualification pathways.

Although the association of science with cleverness may be a potential issue across different subject areas, we suggest that it is particularly exacerbated in the case of science and other high‐status subjects, which are traditionally seen as “hard” subjects. Science's status as an elite subject, “only for the clever,” is not only the result of popular perceptions, but is reproduced institutional practices and technologies of power. For example, in the UK it is common for access to postcompulsory science subjects to be more tightly restricted than for other subjects. In England, students must achieve the top grades of an A or A* grade in the national GCSE examinations at age 16 to be eligible to study academic postcompulsory courses (A levels) in Physics, whereas a lower grade B or C is more common an entry requirement for subjects such as English (DFE, 2012).

Moreover, we have argued that cleverness is a profoundly racialized, classed, and gendered discourse, which is harder for working‐class Black boys and girls to inhabit authentically. The result is that Black students are less likely to imagine themselves following science careers—even though they like science and aspire highly—and that those who do may have to develop considerable resilience to sustain such aspirations over time. It is perhaps little wonder that many Black students choose to aspire otherwise, to jobs within safe, “known” routes. Selena and Vanessa provide encouraging examples of how some young Black women are able to see a place for themselves within science and can imagine themselves into the career of “scientist.” But there is a long way to go. Hanson (2009) found that Black American girls report liking science in high school, often due to strong female role models (often mothers) in their lives, but drift away from science in college.

Our analysis points to several implications for science education: First, we suggest that there is an urgent need to find ways to break the pervasive science = scientist link. Currently policy discourse, and the majority of STEM enrichment activity, prioritizes the science pipeline, encouraging young people to study science as a route to attaining a science career. We suggest that it would be more beneficial to promote the message that science “opens doors” to a wide range of careers at both graduate and technical levels, both in and beyond science. Previous work has, usefully, called for an opening up of the meanings and representations of “scientists” (Scantlebury, Tai, & Rahm, 2007) to enable young people to better relate science to their own life worlds. Our analysis suggests that this could be usefully extended to challenging the science‐scientist link (which is firmly embedded in young people's consciousness), so that science is promoted as not only leading to careers in science, but as also opening up a wide range of potential future careers beyond science. For this, we feel it would be useful to break away from the prevalent metaphor of the “science pipeline,” which we consider to be unhelpful to attempts to widen participation in science. Metaphors can frame our thinking in powerful ways (Lakoff & Johnson, 1980) and the “pipeline” metaphor undermines the value of science for scientific literacy (and the relevance of science education for a wide range of jobs) due to its association of science learning with “training,” in which science education is strongly framed as being primarily a step on the route to a “science job.” We suggest that an alternative metaphor, such as science as a “springboard,” might be more helpful in conveying the value of science qualifications (and scientific skills) for a diverse range of potential future paths.

Second, our analysis calls for challenges to be made to the popular association between science and “braininess.” This means challenging both popular representations of science and scientists but also rethinking social and educational structures that reinforce and perpetuate this relationship. For instance, in the United Kingdom, this would mean ending the practice of entering students for different level examination papers (whereby some can only attain a grade C at most) and creating parity between the sciences and other subjects with regard to entry requirements for advanced‐level courses. Evidence shows that there is also work to be done to ensure equitable pedagogy within science classes (Carlone, 2004), helping teachers and educators to challenge unwitting biases and helping them to provide specific support and encouragement to students from underrepresented groups to enable these students to see science as a potential option for their futures. In the United States, innovative attempts to develop empowering, equitable, and democratic forms of science education, for instance, through the introduction of more culturally congruent and caring approaches to teaching have been shown to raise attainment among Black and other students (e.g., Parsons, 2007; Parsons et al., 2005). Approaches include harnessing the cultural resources and expressive forms of Black students (Elmesky, 2011) and/or the creation of “third spaces” in which to engage disadvantaged urban youth with science and mathematics to support them in their performances of scientific identities and their use of science and math knowledge and skills to transform their lives, both in and out of school (e.g., Tan and Calabrese Barton, 2012; Basu et al., 2011). In these spaces, educators have found ways to value and leverage students’ home knowledge and experiences for science learning, which can help support young people to come to see themselves as “science experts.”

Third, our work highlights the need for a better and fairer (re)distribution of all forms of capital, including science capital, across society. Helping students and families to understand the transferable value of science qualifications could be helpful in inspiring more young people to see science as possible and personally relevant for their own futures. As Alan put it, “Show them like what jobs they can get and [that] you get more money if you do have Science than if you don't.” Our findings suggest that working with families to build science capital may be particularly helpful. This suggestion is supported by wider research. For instance, Calabrese Barton et al.'s (2001) study found that mothers who had spent time doing science with their children were more likely to have a more personal, dynamic, and inquiry‐based view of science. They also found that mothers' perceptions of science were more dynamic when they spoke about situations and contexts that were familiar to them, such as food, nutrition, and child care. In other words, supporting families to feel comfortable and knowledgeable about science and to see its relevance to the lives of parents and children might help more Black students to develop and sustain science aspirations.

In this way, we hope that science educators might help contribute to the social redistribution of economic and cultural and social capital and to help ensure that Black students and families are no longer systematically less privileged in society.

## Footnotes

1 The term “Black” is used here to denote those students in the study who identified as from Black African and/or Caribbean backgrounds. It also includes one student who identified as mixed, with one Black African and one White British parent.

2 See also the literature on stereotype threat, e.g., Steele and Aronson (1995).

3 Cultural capital was determined by responses to items such as parental university attendance (and leaving school before age 16), approximate number of books in the home and frequency of museum visitation. These items were used to provide an overall indication of level of cultural capital. Students in our sample fell into the following categories: very low (2%), low, (23.3%), medium (34.1%), high (20.3%), and very high (20.3%).

4 Of the students who completed the survey there were 50.6% boys, 49.3% girls; 846 (9.1%) in private schools, 8473 (90.9%) in state schools; 74.9% White, 8.9% Asian (Indian, Pakistani, Bangladeshi heritage), 7.5% Black (Black African, Black Caribbean), 1.4% Far Eastern, 7.8% mixed, or other.

5 Of the 5634 students who participated in Survey 2, 2251 (40.0%) were boys and 3358 (59.6%) were girls. (Twenty‐five students did not provide their gender.) A total of 5226 (93%) attended state schools and 408 (7%) attended independent schools. Seven hundred eleven of the 5634 students who completed Survey 2 also completed Survey 1.

6 Subcategories and numbers: White English = 3066; White Scottish = 133, White Welsh = 80, White Irish = 176; Other White = 175; Asian Indian = 213, Asian Pakistani = 156; Asian Bangladeshi = 100, Other Asian = 140, Black Caribbean = 71, Black African = 195, Other Black = 25, Chinese = 42, Japanese = 6, Korean = 13; Other East Asian = 19; Black and White mixed = 112; Asian and White mixed = 92; Black and Asian mixed = 14.

7
http://tisme‐scienceandmaths.org/wp‐content/uploads/2012/06/Mapping‐and‐Classification‐of‐STEM‐Interventions.pdf.

8 Schools tend to select only the highest attaining students as eligible to study for this, the highest status, option for studying science at GCSE.

9 It is not clear from her transcripts, but it is possible that she may have been attracted not just by inherent interest in the programs but popular representations of Black women within them. For example, CSI Miami has a Black female Chief Medical Examiner Alexx Woods (Episodes 1–140) as one of its stars and recurrent black woman doctor in more recent episodes. CSI has some black male stars as does Numb3rs. There is also a famous forensic scientist (one of the “top 10” famous forensic scientists) who is a mixed race African/American woman called Clea Koff, who has written a bestselling book about her years working for the United Nations in Rwanda.

10 Although this may not be the case for all groups of students, for example, see Bøe (2011).

11 For example, see media coverage such as http://www.timeshighereducation.co.uk/176350.article.

12 For example, see http://www.publicsectorexecutive.com/Crime‐reduction/government‐must‐protect‐forensic‐science‐mps.
